# Supplementary material for: BNT162b2 Elicited an Efficient Cell-Mediated Response against SARS-CoV-2 in Kidney Transplant Recipients and Common Variable Immunodeficiency Patients
Source: Viruses. 2023 Jul 30;15(8):1659. doi: 10.3390/v15081659 (PMC10459971; doi:10.3390/v15081659)
Supplement: Supplementary file 1 [file viruses-15-01659-s001.zip › viruses-2517147-supplementary.pdf]

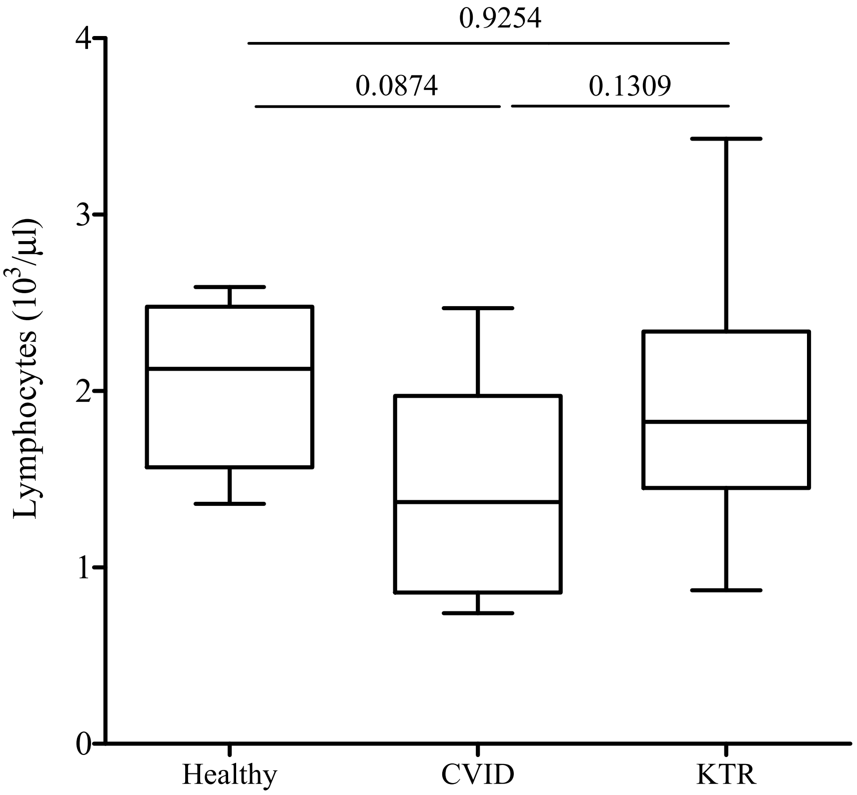


**Supplementary Figure S1.** Lymphocyte count in Common Variable Immunodeficiency (CVID) patients and Kidney Transplant Recipients (KTR) and Healthy donors,. Data are shown as the median (horizontal black line), the 25$^{th}$ and 75$^{th}$ percentiles (boxes) and the 5$^{th}$ and 95$^{th}$ percentiles (whiskers) of 19 CVID, 17 KTR patients and 10 controls.
